# Evaluating the effect of Ramadan Fasting on patients with chronic diseases

**DOI:** 10.12669/pjms.37.4.3199

**Published:** 2021

**Authors:** Guzin Zeren Ozturk, Memet Taskın Egici, Osman Sagsoz, Mulazim Hussain Bukhari

**Affiliations:** 1 Guzin Zeren Ozturk, Associate Professor, Family Medicine Clinic, University of Health Sciences, Sisli Hamidiye Etfal Education and Research Hospital, Istanbul, Turkey; 2 Memet Taskın Egici, Associate Professor, Family Medicine Clinic, University of Health Sciences, Haydarpaşa Numune Education and Research Hospital, Istanbul, Turkey; 3 Osman Sagsoz, Kaynaşlı State Hospital, Düzce, Turkey; 4 Mulazim Hussain Bukhari, Head of Pathology Department, Azra Naheed Medical College, Superior University, Lahore, Pakistan

**Keywords:** Fasting, Ramadan, Non-infectious Disease, Chronic Disease, Consultation

## Abstract

**Objective:**

The study aimed to investigate the effect of Ramadan Fasting on Patients with Chronic Diseases and their experience during fasting.

**Methods:**

This study was a descriptive research that used a researcher-designed questionnaire in face-to-face interviews with individuals who have at least one chronic disease and visit, for any reason, the Istanbul Sisli Etfal Training and Research Hospital polyclinics. We first asked each participant about fasting during Ramadan. If the participant was not fasting, we asked only the reason(s) and collected socio-demographic data. If a participant was fasting, we administered a researcher-designed questionnaire in face-to-face interviews. Our survey consisted of 19 questions that were designed to evaluate the fasting behaviors, current chronic disease and treatment status of individuals, encountered complications during fasting and their socio-demographic data.

**Results:**

The study participants were 253 people (168 females [66.4%]; mean age: 58.06 ± 11,13) with non-infectious diseases. One hundred sixty (63.2%) participants were fasting during Ramadan and 33 of them (20.6%) had consulted a doctor before fasting, 62.5% (n = 100) said they never faced any symptoms during fasting. Most experienced symptom during fasting was fatigue (56.7%; n = 34). A significant relationship occurred between experiencing symptoms while fasting and gender (p = 0.023) and waking regularly for *sahur* (p = 0.029).

**Conclusions:**

Many people with chronic diseases fast and experience symptoms while fasting. Being woman and not waking up for sahur was related with the symptoms during Ramadan fasting. Most participants with NIDs fasted during Ramadan without consulting their doctors.

## INTRODUCTION

Advances in the medical field have increased life expectancy than it was in the earlier times. These advances have led more people to live up to an older age, ultimately giving rise to chronic diseases.^1^ Chronic diseases, also known as non-contagious diseases, are non-infectious, insidious, and slow progressing disorders; however, their complications may cause disabilities. Because non-infectious diseases (NIDs) are still the leading causes of morbidity and mortality globally, they are some of the most important health challenges.^2^ NIDs include cardiovascular diseases, cancers, chronic respiratory diseases, and diabetes. Because of their significant impact on NIDs, nutrition, and lifestyle changes are the cornerstones in the treatment of NIDs.

Many Muslims observe fast during the Islamic month of Ramadan. Ramadan lasts for 29 or 30 days, and fasting begins with *imsak* and ends with *iftar*, which means that people go without eating or drinking anything for 12–20 hours a day depending on where they live. In addition, changes in people’s sleep cycles during Ramadan affect their daily lives. The effects of these changes on their nutrition and lifestyle on NIDs have inspired a great deal of research.

Many studies have shown that fasting does not alter metabolic parameters.^3^,^4^ However, some research works have indicated that it negatively affects blood sugar levels in insulin-using patients with diabetes.^5^,^6^ Even though Muslims whose health may deteriorate because of fasting may be exempt from it, a great number of people with chronic diseases do fast, and some even go against their doctors’ recommendation of refraining from fasting.^7^

Although many studies performed investigated the effects of Ramadan Fasting;^8^ in our study we aimed to investigate the effects of Ramadan Fasting on Patients with Chronic Diseases and their experience during fasting.

## METHODS

We conducted this study with the individuals who come to Istanbul Sisli Etfal Training and Research Hospital polyclinics between August-November 2018 for any of the reasons and who have at least one chronic disease. We defined exclusion criteria of our study as being pregnant, being under 18 years old and not having any chronic diseases. We use the definition of Centers for Disease Control (CDC) about Chronic diseases (CD). CD are defined broadly as conditions that lasts one year or more and require ongoing medical attention or limit activities of daily living or both.^9^ Our study includes diabetes, hypertension, ischemic heart disease, thyroid disease, asthma, chronic obstructive pulmonary disease, cancer, gastritis and other diseases that meet the definition as chronic diseases according to participant information. We first asked each participant about fasting during Ramadan. If the participant was not fasting, we asked only the reason(s) and collected socio-demographic data. If a participant was fasting, we administered a researcher-designed questionnaire in face-to-face interviews. During these interviews, doctors filled the survey. Our survey consisted of 19 questions that were designed to evaluate the fasting behaviors, current chronic disease and treatment status of individuals, encountered complications during fasting and their socio-demographic data. We asked about the symptoms they experienced during fasting like fatigue, headache, hypoglycemia etc. and in response we accepted their statements. As for the hypoglycemia symptom, we questioned the values they measured in their glucose meter. Sisli Etfal Training and Research Hospital Ethics committee approval was received on July 3^rd^, 2018 with reference number 2041.

We entered data into the statistical analysis program SPSS. According to the Shapiro–Wilk test, our study population had abnormal distribution. The socio-demographic data obtained were evaluated with their number and percentage dispersions. Chi-square and t test were used in the statistical analysis. The results were evaluated within the 95% confidence interval, and significance was evaluated at p<0.05

## RESULTS

In total, 253 people with NIDs agreed to participate in this study. A total of 168(66.4%) of them were women, and their mean age was 58.06±11.13. Among the participants, the largest groups were the ones with education levels below high school (73.9%) followed by the ones who earn minimum wage and below (45.5%). The most frequent NID was the hypertension (HT) (75.9%). The diagnosis of HT was correlated with advanced age (p = 0.000). In total, 67.7% of individuals with HT were women.

There were 93(36.8%) people in the cohort who did not fast during Ramadan. Seven (7.5%) of them said that they did not fast because they had different beliefs. Other reasons for not fasting were recommendations against fasting by doctors and the presence of a chronic disease. Of the 33 people who did not observe fast because doctors recommendation not to, 81.8% had HT, 60.6% had diabetes mellitus (DM), and 54.5% had ischemic heart disease (IHD).

**Fig.1:**
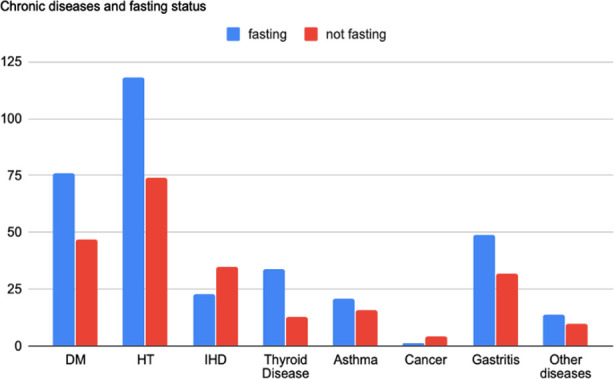
Chronic diseases and fasting status.

Socio-demographic data of the participants were analyzed by dividing them into two groups based on their fasting status (Table-I). Fig.1 presents the chronic disease distribution of individuals who observe fast and who did not observe fast. The most frequent NIDs in the fasting group were thyroid diseases (hypo- or hyperthyroidism), whereas the most frequent NID in the non-fasting group was IHD. Regarding the relationship between fasting and NIDs, the single diagnosis of IHD and not fasting had a significant relationship (p = 0.000).

**Table-I:** Socio-demographic features of fasting and non-fasting individuals.

	Total		Fasting		Non-fasting		P
	N	%	N	%	N	%	
Gender							
Female	168	66.4	108	67.5	60	64.5	0.628
Male	85	33.6	52	32.5	33	35.5	
Education status							
Illiterate	45	17.8	32	20.0	13	14.0	0.482
Below high school	187	73.9	115	71.9	72	77.4	
High school and above	21	8.3	13	8.1	8	8.6	
Income status							
<1500	115	45.5	72	45.0	43	46.2	0.687
1500–3000	105	41.5	69	43.1	36	38.7	
>3000	33	13.0	19	11.9	14	15.1	
Number of chronic diseases							
1	71	28	55	34.4	16	17.2	0.032
2	91	36	54	33.8	37	39.8	
3	60	23.7	34	21.2	26	28	
>4	31	12.3	17	10.6	14	15	
Number of medications used							
<5	169	66.8	117	69.2	52	55.9	0.005
≥5	84	33.2	43	30.8	41	44.1	
Chronic Diseases							
DM	123	48.6	76	61.8	47	38.2	0.641
HT	192	75.9	118	61.5	74	38.5	0.297
IHD	58	22.9	23	39.7	35	60.3	0.000
Asthma	37	14.6	21	56.8	16	43.2	0.376
Thyroid Disease	47	18.6	34	72.3	13	27.7	0.152
Cancer	5	2	1	20	4	80	0.043
Gastritis	81	32	49	60.5	32	39.5	0.534
Other	29	9.5	14	58.3	10	41.7	0.600

Chi-square was used p < 0.05 significant

One hundred sixty (63.2%) participants were fasting during Ramadan. The mean age of the fasting group was lower than the non-fasting group; however, it was not statistically significant (p = 0.750). At the same time, as seen in Table-I, there were no significant relationships between gender, education levels, income status and fasting. The fasting rate decreased with the increase in the number of chronic diseases (p = 0.032). Individuals who take five or more medications daily had a lower rate of fasting (p = 0.005).

Within the fasting group, 20.6% (n = 33) of the individuals had consulted a doctor before fasting. In total, 90% (n = 144) said they woke up every night for *sahur*, 92.5% (148) said they keep taking all their medications during Ramadan and 62.5% (n = 100) said they never faced any symptoms during fasting. When we questioned about the daily water consumption, 32.5% (n = 52) of them answered it was below 1 L, 53.8% (n = 86) said it was 1–2 L, and 3.8% (n = 22) said it was above 2 L. There were no significant relationship between consulting a doctor with waking up for *sahur*, experiencing symptoms during fasting, daily water consumption, and regular medication usage (p = 0.528; 0.289; 0.238; 0.129).

When we divided the participants according to symptom experiencing during fasting, this variable shared no correlation with age (p = 0.151). Table-II shows the relationship between symptom experienced during fasting and other data variables. It was significant only with gender (p = 0.023) and waking up for *sahur* regularly (p = 0.029). With regard to chronic diseases, there was a significant relationship with IHD (p = 0.042) but not with HT (p = 0.115) and DM (p = 0.624).

**Table-II:** Relationship of socio-demographic features and experienced symptoms.

	Not experiencing symptoms during fasting		Experiencing symptoms during fasting		P
	N	%	N	%	
Gender					
Female	61	56.5	47	43.5	0.023
Male	39	75	13	25	
Education status					
Illiterate	18	56.2	14	43.8	0.703
Below high school	74	64.3	41	35.7	
High school and above	8	61.5	5	38.5	
Income status					
<1500	47	65.3	25	34.7	0.784
1500–3000	42	60.9	27	39.1	
>3000	11	59.7	8	42.1	
Number of chronic diseases					
1	34	61.8	21	32.8	0.086
2	37	68.5	17	31.5	
3	23	67.6	11	32.4	
>4	6	35.3	11	64.7	
Number of medications used					
<5	73	62.4	44	37.6	0.963
≥5	27	62.8	16	37.2	
Waking up for sahur					
Everyday	94	65.3	50	34.7	0.029
Sometimes/never	6	37.5	10	62.5	
The daily water consumption					
≤1 L	30	30	22	36.7	0.640
1–2 L	55	55	31	51.7	
>2 L	55	15	7	11.6	

Chi-square was used p < 0.05 significant

When we questioned what kind of symptoms they experienced during fasting, 56.7% (n = 34) of individuals said that they had fatigue, 38.3% (n = 23) of them had headaches, 15% (n = 9) of them had hypoglycemia, 3.3% (n = 2) had fainted, and 6.7% (n = 4) had stomachache. In total, 43.4% (n = 26) of the people had other non-specific symptoms such as hunger, sleeplessness, etc. In total, 19 of 23 individuals who experienced headaches had been previously diagnosed with HT.

**Fig. 2:**
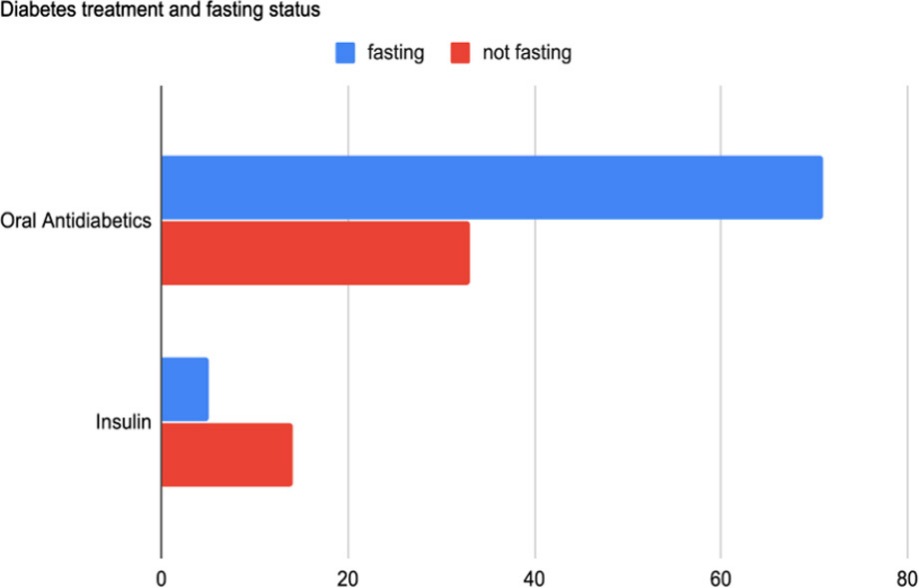
Diabetes treatment and fasting status.

There were 123 diabetic patients and 76 (61.8%) of them were fasting. Fig.2 provides the medication types of fasting and non-fasting patients with diabetes. In total, 6.6% (n = 5) of individuals in the fasting group and 29.8% (n = 14) of individuals in the non-fasting group were using insulin therapy. Thirty (39.5%) DM participants experienced symptoms during fasting. Fatigue was commonly observed in patients with diabetes (n = 20; 66.7%). After that was hypoglycemia; among the patients with diabetes, 8 (10.5%) of them stated that they experienced hypoglycemia, eight of them were using oral anti-diabetics, eight of them did not consult a doctor before fasting and six of them were waking up for *sahur*.

In total, 97 (60.6%) individuals had complaints after iftar. The most frequent one was sleepiness in 65 (67%) individuals. The others were dyspepsia (n = 38, 38%) and constipation (n = 21, 21.6%). Some participants stated more than one complaint. There was no significant relationship between having complaints after *iftar* and age (p = 0.163), education levels (p = 0.278), and income status (p = 0.234), but there was a significant relationship between having complaints after iftar and gender (p = 0.000). Female individuals had complaints after iftar more frequently than males. Participants who experienced difficulty in fasting had complaints after iftar more frequently than others (p = 0.003).

## DISCUSSION

Hypertension (HT) was the most common disease among our participants (75.9%). According to the 2017 Turkey Household Health Research: Risk Factors and Prevalence of Non-Infectious Diseases report, the rate of HT was 16.2%, and it was more common in women.^7^ In the 2010 study TURDEPP 2, the prevalence of HT were 32.3% and 30.9% in women and men, respectively.^10^ In our study, it was more frequent in women. A 2007 study shows that, vein, baroreceptor, and metabolism changes cause increase in blood pressure with aging.^11^ In our study, the diagnosis of HT was correlated with advanced age too.

In total, HT was the most common chronic disease in non-fasting individuals, because doctors recommended against fasting; however, many studies showed that fasting has no effect on blood pressure control.^12^-^14^ It is noteworthy that the majority of those who experienced headache while fasting had been previously diagnosed with HT. This might have happened due to unregulated blood pressure or irregular administration of medication.

Before the start of fasting, Muslims wake up before *imsak* and eat. This practice is known as *sahur*. In a study conducted with healthy individuals in 2006, 66.9% woke up every night for *sahur*.^15^ This rate is 90% in our study, which we believe is because our study was conducted on individuals with chronic diseases. Because the duration of hunger and thirst is prolonged when individuals do not wake up for *sahur*, the status of waking up to *sahur* should definitely be evaluated. Thus, in our study, a significant relationship was found between experiencing symptoms while fasting and waking up for *sahur*.

In our study, we found a significant relationship between IHD and experiencing symptoms during fasting. A 2014 study included patients who had IHD with a normal ejection fraction (EF), and patients who fasted did not experience any new obstruction or chest pain. There was no difference between patients who fasted and those who did not.^16^ We believe this might be due to the independent evaluation of patients from their EFs.

Similar to a 2006 study,^15^ fatigue was the most experienced difficulty in our study. Studies have shown that changes in sleeping and eating patterns affect cortisol regulation during Ramadan,^17^,^18^ and this might be one of the causes of fatigue.

According to studies conducted on fasting and DM, hypoglycemia, hyperglycemia, and ketoacidosis are risks individuals with diabetes, mostly type 1 DM, might experience when fasting.^19^-^21^ While fasting, individuals with diabetes may experience irregularities in blood glucose (BG) levels. A study that monitored the BG levels of patients with diabetes and otherwise healthy people found that there was no significant difference between fasting and non-fasting healthy people, other than a sudden increase of BG right after *iftar*. Moreover, there were differences all day between fasting and non-fasting people with diabetes.^22^ The second most commonly experienced symptom during fasting was hypoglycemia. In this study, 10.5% participants reported hypoglycemia, but in the CREED study, the hypoglycemia rate was 8.8%.^23^ This difference could be due to differences in study participants’ BG regulation status.

American Diabetes Association and International Diabetes Federation have published reports in 2015 and 2016, respectively, about diabetes management during Ramadan.^24^ In regard of these reports’ recommendations, The Turkey Endocrinology and Metabolism Federation has published its own suggestions.^25^ According to suggestions, risk assessment should be conducted one to two months before Ramadan. Patients are asked to self-monitor their BG levels. After the patient’s risk group is determined, suggestions should be made accordingly to guard against complications.

According to our study; many patients with CD experienced symptoms. It is most appropriate to evaluate people by a doctor before fasting, but as seen in our study, the number of people evaluated by a doctor before fasting is low. To prevent this some information can be provided to increase knowledge and consulting doctor of patients with CD.

### Limitations of the study

The study was conducted in a single center and it mostly includes individuals from a specific culture. Part of the data we collected is according to individuals’ statements and not evidence based.

## CONCLUSION

Many people with chronic diseases fast and experience symptoms while fasting. The most experienced symptom was fatigue. Only IHD was related. Also being woman and not waking up for sahur was related too. Most participants with NIDs fasted during Ramadan without consulting doctors. We think that providing written and visual information close to the time of Ramadan fasting and especially reminding that individuals with chronic diseases should consult a doctor will reduce the symptoms they experience due to fasting.

### Authors’ Contribution:

GZO & MTE: Conceived, designed, statistically analyzed, and edited the manuscript, is responsible for integrity of study.

OS: Collected data and wrote the manuscript.

MHB: Reviewed and gave the final approval of the manuscript.
